# Hydroxychloroquine Destabilizes Phospho-S6 in Human Renal Carcinoma Cells

**DOI:** 10.1371/journal.pone.0131464

**Published:** 2015-07-02

**Authors:** Hyung-Ok Lee, Aladdin Mustafa, Gary R. Hudes, Warren D. Kruger

**Affiliations:** Cancer Biology Program, Fox Chase Cancer Center, Philadelphia, Pennsylvania, United States of America; Children's Hospital Boston & Harvard Medical School, UNITED STATES

## Abstract

mTOR inhibitors are used to treat metastatic renal cell cancer (RCC), but most patients eventually become resistant. One possible mechanism for resistance is upregulation of autophagy, a pathway that helps recycle intracellular proteins and promotes cell survival. Hydroxychloroquine (HCQ), a potent autophagy inhibitor used to treat malaria and autoimmune disorders, is currently being studied in the context of cancer treatment. Here, we have investigated the effects of HCQ on three different renal carcinoma derived cell lines. We found that HCQ treatment inhibits RCC cell growth, promotes apoptosis, inhibits mitochondrial oxygen consumption, and increases rates of glycolysis. To understand the molecular mechanism behind these effects, we examined various nodes in the mTOR pathway and compared the effects of HCQ with the effects of the mTOR inhibitor RAD001. A key downstream readout of the pathway, phospho-S6 protein, was inhibited by both HCQ and RAD001. However, the upstream kinase, P70S6K was only inhibited by RAD001 and not HCQ, suggesting that the block by HCQ was downstream of P70S6K. Treatment with the proteasome inhibitor bortezomib restored phospho-S6 levels, suggesting that the reduction of phospho-S6 is caused by increased degradation of phospho-S6, but not total S6. Surprisingly, treatment with other autophagy inhibitors did not exhibit the same effects. Our findings suggest that HCQ causes the down-regulation of phospho-S6 in RCC cell lines via a novel mechanism that is not shared with other autophagy inhibitors.

## Introduction

Renal cell carcinoma (RCC) is the ninth leading cancer killer in men and thirteenth in woman, with over 13,000 deaths in the United States per year [1]. Activation of the mammalian target of rapamycin (mTORC1) pathway is common molecular alteration observed in these cancers [2]. The central player in this pathway is mTOR, a ubiquitously expressed serine/threonine kinase that affects a number of cellular functions, including protein synthesis, cell size and cell proliferation. It also plays a key regulatory role in cell signaling pathways that respond to extracellular and intracellular stimuli, including growth factors, nutrients and energy status [3,4]. MTOR, as part of the mTORC1 complex, has two key targets, P70S6 kinase (P70S6K) and 4E-BP1. Phosphorylation of these targets leads to enhanced protein synthesis [5–7]. Two drugs that inhibit mTOR activation, RAD001 (Everolimus) and CCI-779 (Temsirolimus), are FDA approved for the treatment of advanced renal cell cancer. However, many renal cancers are either intrinsically resistant to the drugs, or become resistant over time [8,9].

Autophagy is an evolutionarily conserved catabolic and homeostatic process that degrades cellular organelles and proteins, helping to maintain cellular biosynthesis during nutrient deprivation, metabolic stress, and hypoxia [10,11]. It involves the formation of double-membrane vesicles, called autophagosomes, which engulf cytoplasmic components and fuse with lysosomes to form autolysosomes. Autophagy’s role in tumorigenesis is complicated, with suppression of autophagy observed in some instances, but activation in others [12]. In laboratory studies, inhibition of autophagy has been reported to enhance the efficacy of a variety of cancer treatments including paclitaxel, 5-flurouracil, radiation, Imatinib mesylate, cyclophosphamide [13–18]. It has also been suggested that upregulation of autophagy is a possible mechanism of resistance to mTOR inhibitors [9,19], and that treatment of cells with mTOR inhibitors can induce autophagy [20,21]. Thus, autophagy inhibitors could potentially overcome resistance to mTOR-targeted therapies for cancer.

Hydroxychloroquine (HCQ), a drug routinely used in the clinical treatment of malaria and autoimmune disorders [22], is a potent inhibitor of autophagy. It prevents lysosomal acidification, thereby interfering with a key step in the autophagic process. In cancer cells, HCQ treatment has been shown to cause increased apoptosis, tumor regression, and delay in tumor recurrence [18,23] effects that support a role for HCQ in clinical cancer therapy. Clinical trials investigating the potential benefit of adding an autophagy inhibitor to standard treatment are ongoing in several tumor types [11,24].

In this study, we have examined the effect of HCQ treatment on renal cancer derived cell lines. We found that HCQ was an effective growth inhibitor of human RCC cell lines, inhibiting growth, promoting apoptosis, and effecting cellular metabolism. Surprisingly, we also found that HCQ altered the levels of the mTORC1 activation marker phospho-S6 and that this effect was mediated by a different mechanism than that observed for the mTOR inhibitor RAD001. Our findings suggest that HCQ may be useful in the treatment of RCC.

## Materials and Methods

### Cell lines, culture conditions, and reagents

Human RCC cell lines, characterized as an adenocarcinoma (ACHN) and clear cell carcinomas (Caki-1 and 769-P), were originally purchased from ATCC [25–27]. All cells were cultured in RPMI with 10% FBS, 1% glutamine, and 1% Pen/Strep. For *in vitro* experiments, cells were seeded on the appropriated plates overnight and treated with HCQ (75 or 100 μM, Acro Chemicals), RAD001 (10 μM, LC Laboratories), bafilomycin A1 (50 nM, Sigma), or spautin-1 (10 μM, Sigma) for 48 hours. Bortezomib (Velcade, 1 μ g/ml) was obtained from the Fox Chase Cancer Center pharmacy and treated cells for 16 hours.

### Cell lysates and Western blotting

Cell lysates were prepared in M-PER lysis buffer (Thermo Scientific) supplemented with protease inhibitors (Roche). Twenty μg of cell lysate was separated by 4–12% SDS-PAGE (Invitrogen) under reducing conditions and transferred to nitrocellulose. The membranes were probed with antibodies using SNAP i.d. system (Millipore) and developed by enhanced chemiluminescence (Thermo Scientific). When the same samples required analysis by different antibodies, blots were stripped using Restore Plus Western Blot Stripping Buffer (Thermo Scientific) and re-probed. The following antibodies were used: S6, P-S6 (ser235/236 or ser240/2440), P70S6K, P-P70S6K (ser371 or thr389), P-PP1 (thr320), p62, 4EBP1, p-4EBP1 (ser65 or thr30), anti-rabbit IgG (Cell Signaling Inc.); PP1 (Santa Cruz Biotechnology); LC3 (Novus Biologicals); actin (Sigma); and anti-mouse-IgG (Amersham Bioscience). Gel images were captured and quantified using the FluorChem SP system (Alpha Innotech). Each experiment was done with at least 3 independently isolated protein extracts. The relative intensities of each protein are assigned as Arbitrary Units obtained by calculating the ratio of test sample divided by a control. The specific activity of the kinases was calculated as the ratio of the scanned optical densities of the phosphorylated forms divided by total expression levels (P/T). The ratios of each LC3(II) relative to the LC3 (I) were calculated by densitometric analysis to monitor the autophagy flux.

### *In vitro* kinase assay and immunopricipitation

For *in vitro* kinase assays with purified proteins, recombinant S6 protein (210 ng, Abnova) and recombinant active P70S6K (100 ng, R&D systems) were incubated in 1x kinase buffer (Cell Signaling Inc.) with various amount of HCQ or RAD001 in the presence (25 μM) or absence of ATP for 30 minutes at 30°C. Total and phosphorylated S6 at ser235/236 and ser240/244 were detected by western analysis using phosphospecific antibodies (see previous section). Note that recombinant GST-tagged S6 (53 kd) is distinguished from endogenous S6 (32 kd) on the western blot.

For immunoprecipitation kinase assays, ACHN cells were either untreated, treated with RAD001 (10 μM) or HCQ (100 μM) for two days, and extracts were prepared as described above. Cell lysates (525 μ g) were incubated with mouse monoclonal P70S6K antibody (2 μ g, Santa Cruz Biotechnology) overnight at 4°C. Protein A/G beads (20 μl, Santa Cruz Biotechnology) were added, and the mixture incubated for four hours at 4°C. Protein was recovered by centrifugation, pellets were washed four times with M-per cell lysis buffer, and once with 1x kinase buffer. IP pellets were suspended into 40 μl of kinase buffer and kinase reactions were set up as described above, using 20 μl of immunopreciptiated P70S6K per assay. A negative control consisted of Protein A/G beads without antibody incubation.

For the P-S6 immunoprecipitation assay, ACHN cells were either untreated, treated with RAD001 (10 μM), HCQ (100 μM), bortezomib (1 μg/ml), along or in combination for 16 hours. Cell lysates (200 μg) were incubated with rabbit P-S6 antibody (ser240/244, 1 μg/ml, Cell Signaling) overnight at 4°C. Immune complexes were prepared as described above and analyzed by western analysis using mouse ubiqutin antibody (Santa Cruz Biothechnology) or mouse P-S6 antibody (ser240, Dako).

### Immunofluorescence staining

RCC cells (5,000) were plated in 8-well chamber slides overnight and treated with HCQ (75 μM) or RAD001 (10 μM) for 2 days. Cells were fixed with 4% paraformaldehyde, permeabilized with 0.1% TritonX-100 in PBS, and stained with following antibodies; rabbit anti-phospho S6 (Ser235/236, Alexa Fluor 488 conjugated) (Cell Signaling Technology), mouse anti-S6 (Cell signaling Technology), mouse anti-phospho-P70S6K (Thr389) (Millipore), and mouse anti-P70S6K (Santa Cruz Biotechnology). Alexa Fluor 647 conjugated anti-mouse IgG antibody (Life Technology) was used for secondary antibody. Nuclear DNA was visualized using a DAPI mounting solution (Vector Laboratories).

### Cell proliferation and apoptosis assays

Cells (1x10^4^) seeded in a 96-well plate for overnight were treated with HCQ, RAD001, bafilomycin A1 (50 nM), or spautin-1 (10 μM) for two days. MTT reagent (3-(4,5-dimethylthiazolyl-2)-2,5-diphenyltetrazolium bromide, Promega) was added for an hour before spectrophotometric measurement at 490 nm. Each measurement was made in triplicate, and the average value was calculated. The assay was performed on three biological replicates.

For apoptosis assays, cells were seeded overnight and treated with HCQ and RAD001 as described above. Cells were then harvested and stained with Annexin V-PE and 7-AAD (PM2) using a Guava Nexin kit (Millipore). Non-apoptotic (annexin V- and 7-AAD-), early apoptotic (annexin V+ and 7-AAD-), and late apoptotic (annexin V+ and 7-AAD+) cells were measured by Guava System (Millipore), and the measurement was analyzed by CytoSoft software (Millipore).

### Lentivirus-mediated knockdown

pLKO.1-derived vector with shRNA targeting human atg7 (TRCN0000007587) was purchased from Sigma. Virus was produced using a second-generation packaging system in 293T cells as according to manufacturer’s instructions. ShRNA against GFP was generously provided by Dr. D. Connelly at FCCC and served as a negative control. Because the lentiviral shRNA particles also encode a puromycin resistance gene, the cells were selected in culture medium containing puromycin (4 μg/ml, Sigma) for two weeks for stable transduction.

### Metabolic analysis by XF96 Extracellular Flux Analyzer

A Seahorse Flux Analyzer was used to measure both the oxygen consumption (OCR) and extracellular acidification rate (ECAR). RCC cells were seeded in XF96 microplate (10,000 cells/well for ACHN and Caki-1, 6,000 cells/well for 769-P) and treated with HCQ (75 μM) overnight. The cells were grown in assay buffer (DMEM base supplemented with 31 mM NaCl, 1x GlutaMax, 10 mM pyruvate, 25 mM glucose, pH 7.4, Sigma) for one hour and analyzed in the Seahorse XF96 Extracellular Flux Analyzer, first under basal conditions, and then followed by sequential injection of four pharmacologic agents affecting respiration and glycolysis: (1) oligomycin (1 μM), (2) carbonyl cyanide 4-(trifluoromethoxyphenylhydrazone (FCCP, 300 nM), (3) 2-Deoxy-D-glucose (DG, 100 μM); and (4) rotenone/antimycin (1 μM each). After the assay had been done, cells in plate were used to determine protein concentration by the BCA protein assay (Thermo Scientific), and metabolic data were normalized to protein concentration. Each experiment was done in 16 wells.

Basal OCR was calculated by subtracting the mean of the last three time points (after rotenone/anitmycin) from the mean of the first three time points. ATP-driven OCR was determined by subtracting the mean of the three time points after oligomycin treatment from the first three time points. Maximal respiration was determined by subtracting the mean of the last three time points from the mean of the six time points after FCCP addition. Basal ECAR was calculated by subtracting the mean of the last three time points (after 2-DG addition) from the mean of first three time points. Glycolytic reserve capacity was calculated by subtracting the first three time points from the mean of the three time points after oligomycin addition.

### Statistical Analysis

Values are expressed as mean + SD. We used ANOVA or t-test to compare group differences using Prism software (GraphPad Software). A p-value of 0.05 was considered significant. In each graph, error bars show standard deviation, and the letter at top indicates statistically significant differences between columns with different letters (P<0.05, ANOVA with Tukey post-hoc test).

## Results

### HCQ triggers apoptosis and suppresses cell proliferation

**Fig 1:**
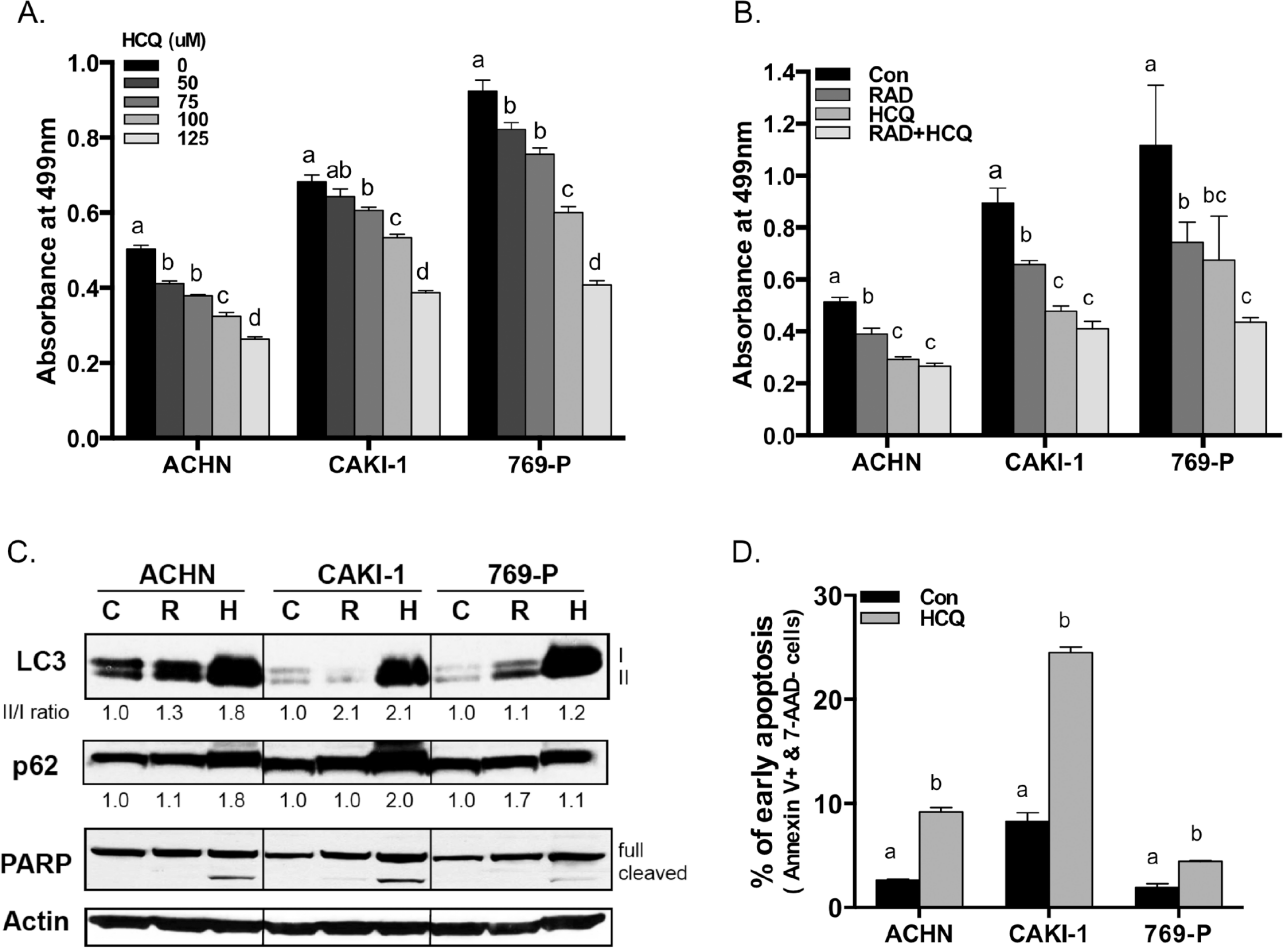
HCQ effects on cell proliferation and apoptosis. **(A)** Three different human RCC derived cell lines were seeded on 96-well plates at a concentration of 1x10^4^ cells/well in triplicate and were treated with HCQ from 0 to 125 μM. After 48 hours, cell growth was measured using an MTT assay. Error bars show standard deviation, and the letter at top indicates statistically significant differences between columns with different letters (P<0.05, ANOVA with Tukey post-hoc test). **(B)** Indicated cell lines were incubated with either nothing, RAD001 (10 μM), HCQ (75 μM) or combination of RAD001 and HCQ for two days. Growth was measured as in (A). **(C)** RCC cells were cultured in the absence (C) or presence of either 10 μM RAD001 (R) or 75 μM HCQ (H) for two days, and Western analyses was used to measure the levels of autophagy (p62, LC3) and apoptosis (PARP) markers. The different forms of LC3 and PARP are shown. The relative intensities of each protein are assigned as Arbitrary Units obtained by calculating the ratio of test sample divided by a control. **(D)** Indicated cell lines were treated with either nothing or 75 μM HCQ for 48 hours and then assessed by staining and cell sorting for the apoptotic markers Annexin V-PE and 7-AAD. Bars represent the percentage of early apoptotic cells in each sample.

We initially examined the effect of HCQ treatment on cell growth of three different RCC derived cell lines (Fig 1). We found that HCQ inhibited growth of all three lines in a dose dependent manner. At the highest concentration of HCQ, cell growth inhibition was similar between all three lines, ranging between 43 and 56%. We also compared the effects of HCQ, the mTOR inhibitor RAD001, and the combination of both drugs on cell growth (Fig 1B). We found that growth inhibition by 75 μM HCQ was similar to that induced by 10 μM RAD001. However, the combination of HCQ and RAD001 together did not exhibit statistically increased growth inhibition compared to either drug alone. To confirm that HCQ was inhibiting autophagy, we examined the status of two autophagy markers, LC3 and p62. Treatment with HCQ resulted in a significant increase in the levels of both LC3(II) and p62, indicating that a late-step in autophagy was being inhibited. (Fig 1C and S1 File). We also measured the levels of apoptosis marker PARP in HCQ treated cells and found that HCQ caused PARP cleavage (Fig 1C). Lastly, flow cytometry showed that HCQ treated cells had an increased number of Annexin+ /7-AAD- cells (Fig 1D). Taken together, these data show that inhibition of autophagy by HCQ triggers apoptosis and suppresses cell proliferation in RCC cell lines.

### Dysregulation of mitochondrial functions by HCQ

**Fig 2:**
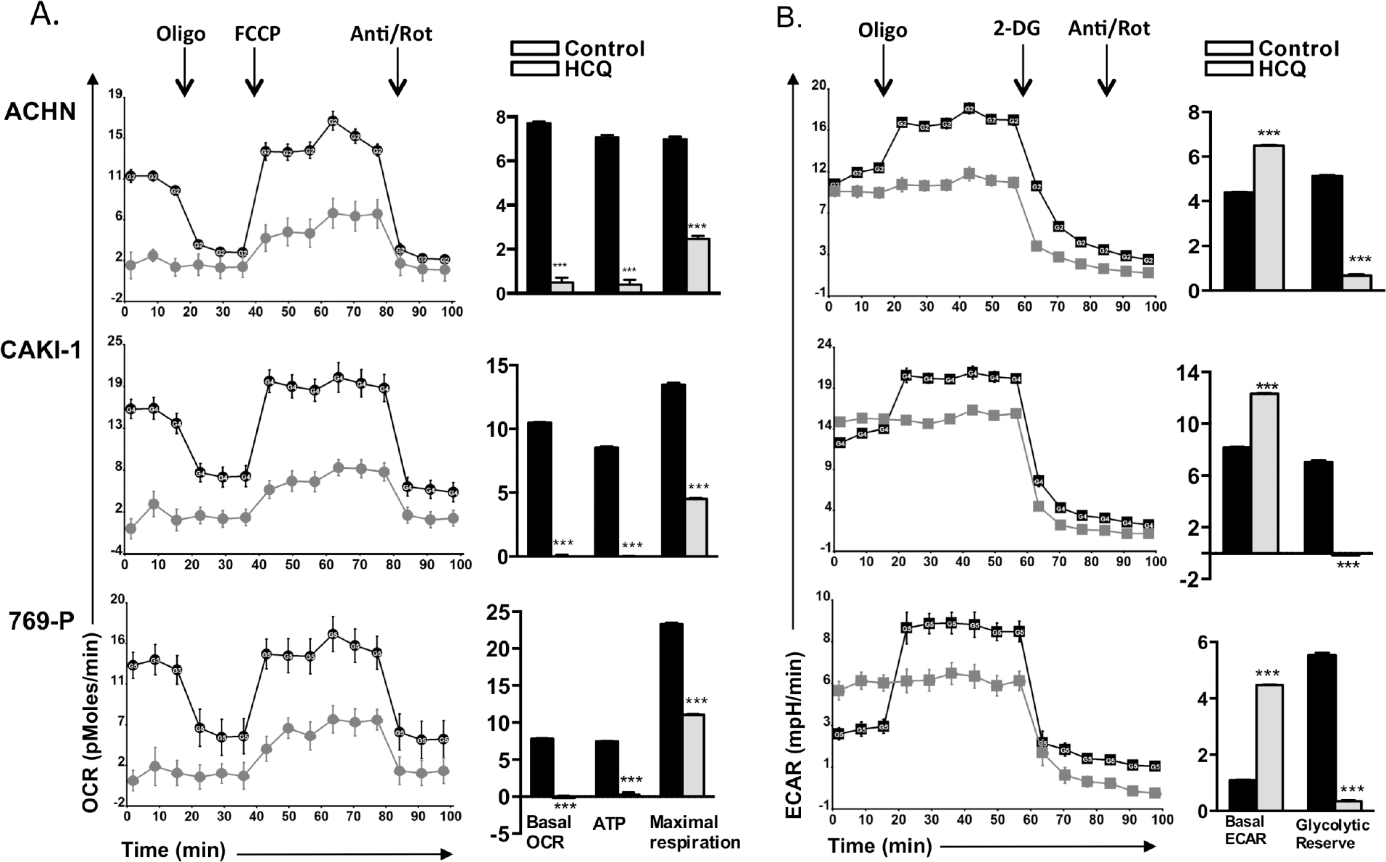
HCQ effects on respiration and glycolysis. RCC cells were incubated in the absence (control) or presence of HCQ (75 μM) overnight, and the cells were then tested for oxygen consumption using a Seahorse XF96 Analyzer. **(A)** The respiration assay examines oxygen consumption in the presence of four pharmacologic agents that affect mitochondrial function. These agents are oligomycin (1 μM), FCCP (300 nM), 2-DG (100 μM), and antimycin A/rotenone (1 μM each). The left side of the figure shows the actual OCR consumption measured at each time point. All experiments were done in 16 wells and error bars show standard deviation. On the right is a bar chart showing the derived basal OCR, ATP synthesis, and maximal respiration (see Methods). Asterisks indicates difference from controls at p<0.001. **(B)** Glycolysis rates were determined by measuring lactate formation. Cells were grown in the presence of 25 μM glucose for 1 hour. Oligomycin was injected to inhibit mitochondrial ATP production and 2-DG was added to determine background levels of glycolysis. Basal ECAR and glycolytic reserve capacity was determined as described in methods.

One important function of autophagy is to recycle damaged mitochondria [28]. Therefore, if autophagy was inhibited, it could lead to alterations in cellular energy metabolism. To test this, we measured both mitochondrial respiration and glycolytic rates in RCC cells using a Seahorse XF analyzer. We found that HCQ-treatment in all three cell lines resulted in a marked reduction in the basal oxygen consumption rate (OCR) (Fig 2). Treatment with the ATP synthase inhibitor Oligomycin relieved much of this difference, indicating that the increased O_2_ utilization in untreated cells was due to mitochondrial driven ATP production. The OCR after treatment with the mitochondrial uncoupling agent FCCP, revealed that HCQ treated cells have significantly reduced maximal mitochondrial respiration, suggesting that the cells have fewer functioning mitochondria. Conversely, HCQ-treated cells show enhanced glycolysis, as indicated by high basal extra-cellular acidification rate (ECAR) (Fig 2B). However, no significant change was observed in ECAR by HCQ after oligomycin administration, indicating that HCQ significantly diminished the glycolytic reserve capacity. Taken together, these results show that HCQ reduces mitochondrial oxidative respiration, reduces ATP production, and maintains higher basal ECAR. These findings are consistent with the idea that inhibition of autophagy causes the accumulation of damaged mitochondria which results in reduced ATP biosynthesis. To compensate, HCQ treated cells have to increase glycolysis to maintain intracellular energy homeostasis.

### HCQ reduces phosphorylation of S6 downstream of mTORC1

**Fig 3:**
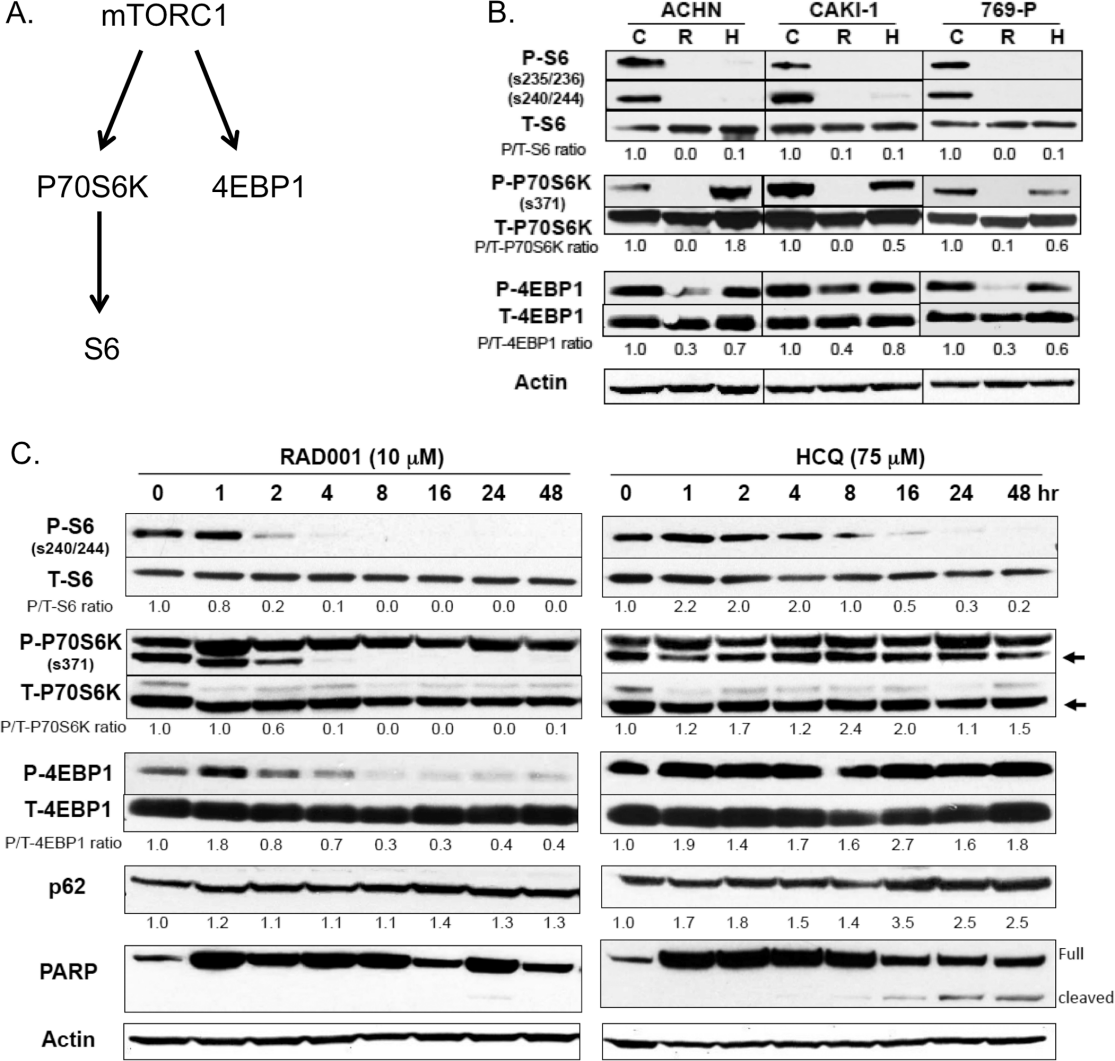
HCQ regulates the phosphorylation of S6 by mTOR-independent pathway. **(A)** Simplified overview of mTORC1 signaling pathway. **(B)** RCC cells were cultured in the absence (control) or presence of RAD001 (10 μM) and HCQ (75 μM) for two days. Western blot analyses were used to determine the level of total and phosphorylated forms of ribosomal protein S6, P70S6K, and 4EBP1. Actin is also shown as a loading control. **(C)** 769-P cells were treated with either RAD001 or HCQ for the indicated amount of time and examined for the indicated proteins. The arrow shows the proper band for the phosphorylated form of P70S6K. (Upper band is cross-reacting band of unknown identity).

We next compared the effect of HCQ and RAD001 on the mTORC1 pathway. There are two major downstream readouts of this pathway: 1) phosphorylation of 4EBP1, and 2) phosphorylation of ribosomal protein S6 (Fig 3). 4EBP1 is a direct target of mTORC1, while S6 phosphorylation is indirect, proceeding via P70S6 kinase. S6 phosphorylation occurs on two pairs of serine residues, ser235/236 and ser240/244. Consistent with the literature, RAD001 treatment caused complete inhibition of S6 phosphorylation on both pairs of residues, inhibited phosphorylation of ser371 of P70S6K, and inhibited 4EBP1 phosphorylation in all three cell lines (Fig 3B). In contrast, HCQ treatment caused inhibition of S6 phosphorylation, but had no significant effect on the levels of phospho-P70S6K or phospho-4EBP1. This experiment indicates that HCQ affects levels of phospho-S6 protein at some step downstream of P70S6K.

To explore this further, we performed a time course experiment in which 769-P cells were exposed to either RAD001 or HCQ for 0–48 hours (Fig 3C). Maximal inhibition of S6 phosphorylation by HCQ was not achieved until 16–24 hours, at about the same time as upregulation of the autophagy marker p62 and cleavage of PARP was first observed. LC3(II) accumulation was first observed at 2 hours and highly induced by 16 hours, indicating high autophagic flux in HCQ-treated cells (S2 File). In contrast, RAD001 inhibition of S6 phosphorylation occurred by four hours and was accompanied by reduced P70S6K and 4EBP1 phosphorylation. These findings show that HCQ and RAD001 affect phospho-S6 levels by different mechanisms.

To investigate whether these effects are specific to HCQ, we tested two other autophagy inhibitors, bafilomycin A1 and spautin-1. Bafilomycin A1, like HCQ, works by inhibiting acidification of the lysosome before it merges with the autophagosome [29]. Spautin-1 works at an earlier step in the autophagy pathway, causing increased degradation of the key autophagy regulator Beclin 1 [30]. We found that, in contrast to HCQ, treatment of cells with either of these agents resulted in downregulation of the mTORC1 pathway as judged to decreased levels of P-S6, P-P70S6K, and P-4EBP1 (Figure A in S3 File). We also found that these inhibitors inhibited cell growth to a similar extent as HCQ (Figure B in S3 File). These findings imply that HCQ affects phospho-S6 levels in a different mechanism than these other autophagy inhibitors.

### Atg7 knock-down does not mimic HCQ in RCC cell lines

**Fig 4:**
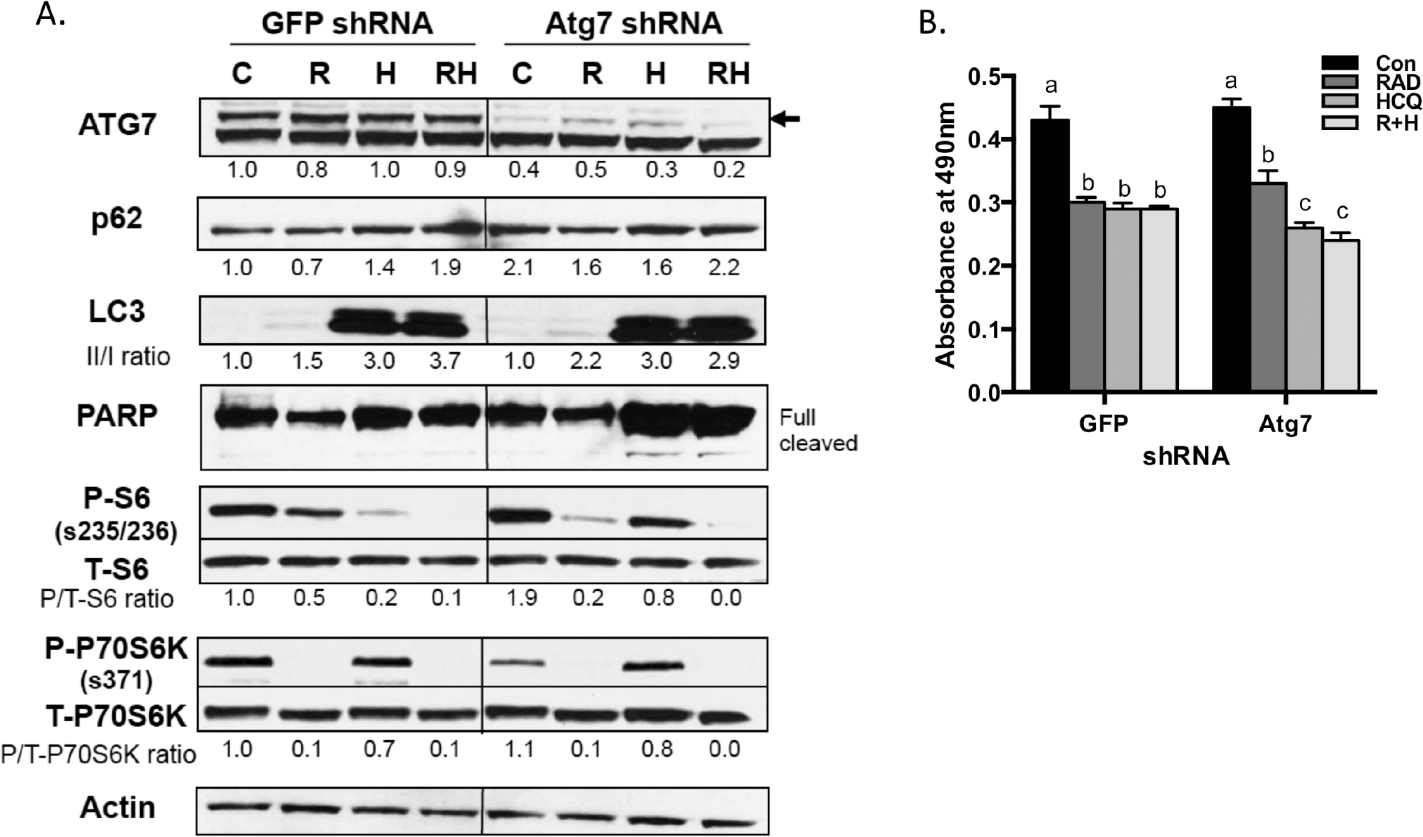
Atg7 is not required for S6 phosphorylation in RCCs. **(A)** 769-P cells were infected with lentivirus, encoding either a control GFP shRNA or the essential autophagy regulator Atg7 shRNA. Stably transfected cells were grown in the presence or absences of drugs for two days. Western blot showed the efficiency of ATG7 knockdown and expression of the selected protein markers. Arrow indicates ATG7 band. **(B)** MTT assay was performed with shRNA-transfected 769-P cells (5x10^3^/well) at two days following drug treatment. Error bars show standard deviation, and the letter at top indicates statistically significant differences between columns with different letters (P<0.05, ANOVA with Tukey post-hoc test).

Atg7 encodes a protein that plays a key role in autophagy and is required for the fusion of peroxisomal and vacuolar membranes. Knockdown of Atg7 in melanoma cells impairs cell growth and induces cell death [21]. To examine whether Atg7 depletion acts similarly in RCC cells, we introduced shRNA against Atg7 into 769-P cells and determined its influence on autophagy, apoptosis, S6 phosphorylation, and cell proliferation. Western analyses confirmed that the level of Atg7 protein was markedly decreased in cells infected with Atg7 shRNA compared to cells infected with control GFP shRNA (Fig 4). With regards to autophagy markers, Atg7 depletion caused a modest increase in p62 levels and no significant increase in LC3 (Fig 4A, compare two lanes labeled “C”). However, given that Atg7 is required relatively early in the process of autophagosome formation, this is not unexpected as the autophagosomes do not form [12]. There were no effects on PARP cleavage, phospho-S6 levels, or differences in cell proliferation (Fig 4B). We also examined the effects of combining Atg7 depletion with HCQ, RAD001, or both together. Atg7-depleted cells treated with RAD001 behaved quite similarly to non-depleted cells with regards to all the markers examined. However, HCQ or combination with RAD001 treatment to Atg7-depleted cells enhanced apoptosis shown as increased cleaved PARP level and inhibited cell proliferation (Fig 4B). Our data indicate that Atg7 depletion does not mimic HCQ treatment, but Atg7 depletion with HCQ treatment enhanced inhibition of cell growth in 769-P cells.

### HCQ reduces phospho-S6 levels by a proteasome-mediated process

**Fig 5:**
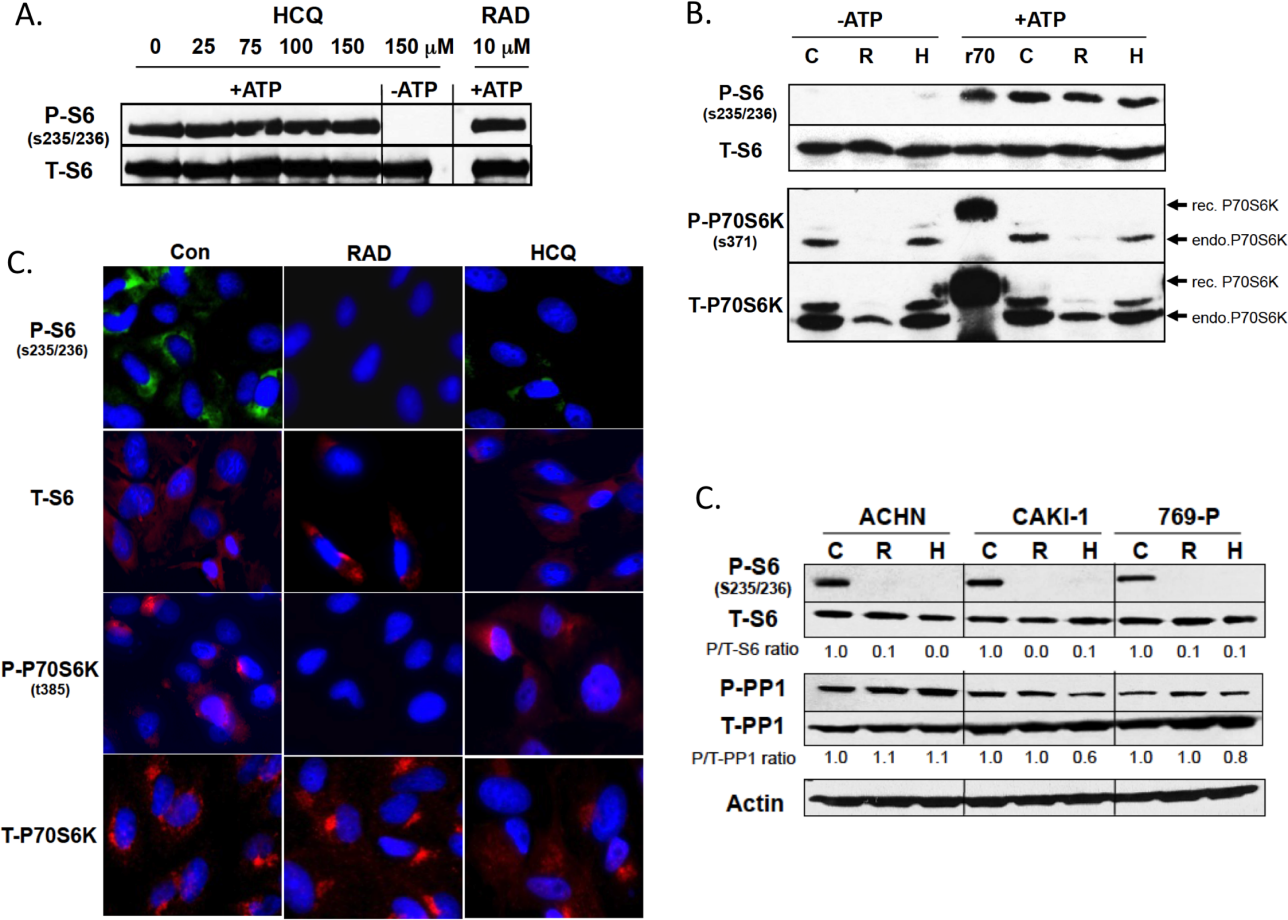
Effects of HCQ on P70S6K and PP1 activity and localization of S6 and P70S6K. **(A)** *In vitro* kinase reaction of purified recombinant P70S6K was performed as described in Methods. Levels of phospho and total S6 determined by western blot. Number on top shows amount of HCQ added. Lane labeled RAD is reaction in which 10 μM RAD001 with ATP. **(B)** *In vitro* kinase activity was performed on immunoprecipitated P70S6K. Extracts were made from either control (C), RAD001 (R), or HCQ (H) treated ACHN cells. Lanes on left show reactions without added ATP. Lane labeled r70 contains recombinant epitope tagged P70S6K, which runs slightly slower than endogenous protein. Reaction mixture was tested for phospho-S6, total S6, phospho-P70S6K, and total P70S6K **(C)** Immunofluorescence of 769-P cells either treated with nothing, RAD001 or HCQ and stained with indicated antibody. Nuclear DNA was visualized by DAPI. **(D)** Cells of the indicated type were untreated or treated with RAD001 or HCQ. Extracts were then examined for phospho-S6, total S6, phospho-PP1 or total PP1.

We next performed a series of experiments designed to understand the mechanism by which HCQ could reduce phospho-S6 levels in the presence of normal amounts of phospho-P70S6K. We first tested the idea that HCQ might interfere with S6 phosphorylation by directly inhibiting phospho-P70S6K activity. Therefore, we added HCQ to an *in vitro* kinase reaction containing purified phospho-P70S6K and S6 protein (Fig 5). We observed no effect of HCQ on S6 phosphorylation even when added to a maximum of 150 μM, twice the concentration used in our cell line studies. Control reactions included leaving out ATP, which is required for S6 phosphorylation, and the addition of RAD001, which had no effect. These findings indicate that HCQ did not directly interfere with phospho-P70S6K activity.

Another possibility was that HCQ might alter post-translational modifications of P70S6K and that this might make it enzymatically inactive. To test this, we immunoprecipitated P70S6K from extracts derived from either untreated, RAD001, or HCQ treated cells, and then examined their ability to phosphorylate recombinant S6 in an *in vitro* kinase reaction (Fig 5B). The levels of P-S6 were identical in all three extracts tested. Controls included leaving ATP out of the reaction mixture and performing the *in vitro* reaction with purified recombinant His-tagged P70S6K. The results show that there is no difference in the *in vitro* enzymatic activity of immunoprecipitated P70S6K from any of the extracts, indicating that the P70S6K present in HCQ treated cells lacks any sort of post-translational modification that would make it less enzymatically competent. An unexpected finding from this experiment was that S6 phosphoryation occurred in extracts from RAD001 treated cells despite the lack of phosphorylation of the S371 residue of P70S6K, suggesting that phosphorylation of S371 is not critical *in vitro*.

We next hypothesized that HCQ might be affecting the *in vivo* localization of S6 or P70S6K. We stained HCQ, RAD001, and untreated ACHN cells with antibodies directed against S6, phospho-S6, P70S6K, and phospho-P70S6K, and visualized the cells using immunofluorescence. No obvious differences in the staining patterns of either total S6 or P70S6K in treated vs. untreated cells were observed (Fig 5C). However, consistent with our Western results, we were able to confirm the lack of phospho-S6 and phospho-P70S6K in RAD001 treated cells and the lack of phospho-S6 in HCQ treated cells. These results suggest that altered intracellular localization is not the explanation for HCQ’s inhibition of S6 phosphorylation.

Another possibility was that the difference in phospho-S6 levels might be related to differences in active phosphatase 1 (PP1), which controls dephosphorylation of S6 [31]. PP1 is regulated by its own phosphorylation, with the phosphorylated form being inactive. Western blot analysis using anti-bodies directed against total PP1 and phospho-PP1 did not reveal any significant difference in phospho-PP1 or total PP1 levels in any of the treated or untreated cells (Fig 5D), suggesting this was not the explanation.

**Fig 6:**
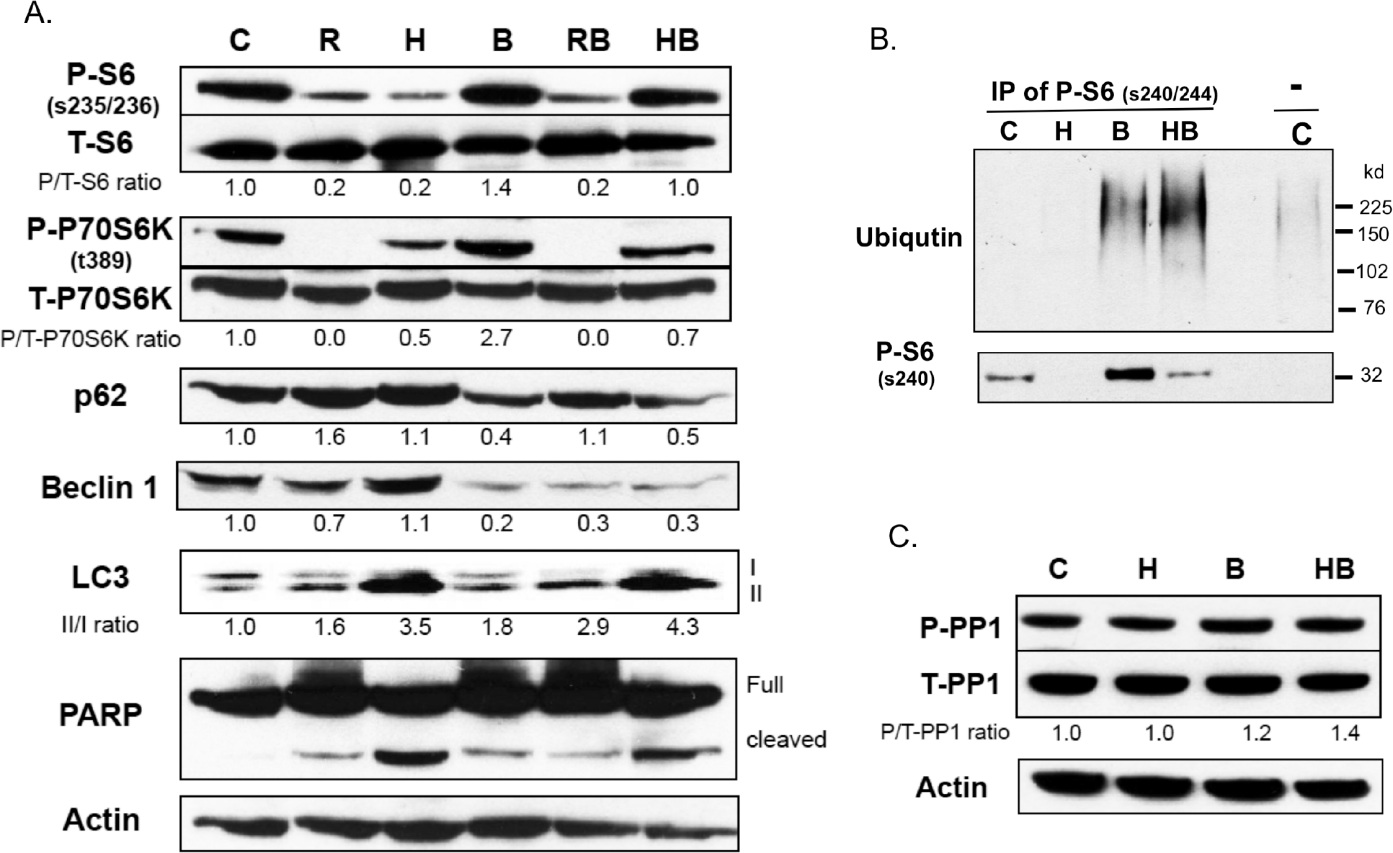
Effect of bortezomib on RAD001 and HCQ treated cells. **(A)** ACHN cells were treated with either nothing (C), 10 μM RAD001 (R), 100 μM HCQ (H) with or without added bortezomib (B) for 16 hours. Extracts were then analyzed by Western blot for phospho-S6, S6, phospho-P70S6K, P70S6K, p62, beclin 1, LC3, PARP, and actin. **(B)** For pull-down assay**,** ACHN cell lysates as described in (A) were incubated with rabbit P-S6 antibody (ser240/244, 1 mg/ml) overnight at 4°C. Immune complexes were analyzed by western analysis using ubiqutin antibody and mouse P-S6 antibody (ser240). **(C)** ACHN cell lysates were analyzed by Western blot for phospho-PP1 and total PP1.

The final hypothesis that we entertained was that HCQ might specifically affect the stability of phospho-S6 relative to the unphosphorylated S6. Therefore, we compared the levels of phospho-S6 and total S6 in control, RAD001, and HCQ treated cells in the presence and absence of the proteasome inhibitor bortezomib. We found that bortezomib treatment had no effect on the levels of phospho-S6 in untreated and RAD001 treated cells, but resulted in a large increase in phospho-S6 levels in HCQ treated cells (Fig 6). We did not observe any difference in total S6 levels, suggesting that phospho-S6 is subjected to proteasome mediated degradation in HCQ treated cells. Bortezomib had no effect on either total S6, total P70S6K, phopho-P70S6K, p62, LC3, or actin levels, indicating that the effect is specific to phospho-S6.

To prove that ubiquitin-mediated proteolysis is involved in phospho-S6 clearance, we immunoprecipitated phospho-S6 and immunoblotted with a polyubiqutin antibody to detect ubiqutinated proteins (Fig 6B). Treatment with bortezomib resulted in the accumulation of polyubiqutinated phospho-S6 and the amount of ubiquitinated phospho-S6 was increased by co-treatment with HCQ. These findings are consistent with the hypothesis that HCQ increases ubiquitination of P-S6 resulting in its degradation by the proteasome. In addition, neither HCQ, bortezomib, nor the combination of both affected the levels of phospho-PP1 or total PP1 (Fig 6C), indicating that bortezomib is not working indirectly by modulation of phosphatase activity.

## Discussion

In the studies described here, we have examined the effects of the HCQ on three different RCC derived cell lines. We found that HCQ inhibited cell growth, induced apoptosis, and altered cellular energy metabolism in all thee lines. With regards to energy metabolism, we found that HCQ suppressed respiration, but enhanced glycolysis. Our findings are consistent with previous work indicating that lysosomal membrane permeablization caused by HCQ results in mitochondrial membrane permeablization due to Bax insertion and subsequent apoptosis [32]. The observed elevation in glycolysis is most likely a compensatory response for the cells to maintain ATP levels.

The rationale behind these studies was the idea that HCQ, a known inhibitor of autophagy, might synergistically interact with inhibitors of the mTOR pathway that are used in the clinic to treat advanced RCC. Specifically, it has been proposed that upregulation of the autophagy pathway might allow cell survival in the presence of mTOR inhibition by increasing recycling of proteins [33]. However, we did not see strong evidence for synergy between the mTOR inhibitor RAD001 and HCQ. Our findings are in contrast with those reported by Bray et al., in which they showed significant synergy between chloroquine (CQ) and the mTOR inhibitor CCI-779 in several different RCC cell lines [34]. Our finding that ATG7 knockdown failed to mimic HCQ effects is also different. There are several possible explanations for these discrepancies including differences in cell lines, differences between CQ and HCQ, and differences between RAD001 and CCI-779. Understanding these differences may be important in the design of clinical trials involving the use of HCQ to treat cancer.

Our most interesting finding was that HCQ treatment resulted in reduced phospho-S6 levels. S6 protein is a component of the 40S ribosomal subunit, and genetic studies suggest that phosphorylation of S6 plays an important role in controlling cell size and protein synthesis [6]. In addition, levels of phospho-S6 are a frequently used marker of activation of the mTORC1 pathway [2]. We found that HCQ treatment resulted in down regulation of phospho-S6 levels, but not phospho-4EBP1 levels or phospho-P70S6K levels. This was in contrast to the effects of RAD001, bafilomycin A1, and spautin-1 on these same cells, which inhibited phosphorylation of all three molecules. These findings suggest the reduction of phospho-S6 levels in HCQ treated cells involves a unique mechanism not found in all autophagy inhibitors.

To understand how HCQ caused reduction in phospho-S6 in the presence of normal levels of phospho-P70S6K, we explored a variety of hypotheses. We showed that HCQ did not directly interfere with P70S6K activity and that phospho-P70S6K immunoprecipitated from HCQ treated cells was competent to phosphorylate S6 *in vitro*. These results show that the phospho-P70S6K from HCQ treated cells was enzymatically functional. We also did not observe any effect of HCQ on either the subcellular localization of S6 or P70S6K. There was also no difference in either the levels or the phosphorylation status of PP1, the phosphatase for S6. However, we did find that treatment with the proteasome inhibitor bortezomib restored phospho-S6 to levels similar to those found in untreated cells. Since we did not observe any difference in the levels of non-phosphorylated S6 or several other proteins that were examined (see Fig 6A and 6C), we believe these effects are specific and do not represent general effects of proteasome inhibition by HCQ. Therefore, these findings suggest that HCQ causes decreased phospho-S6 levels by increasing the rate of its degradation via the proteasome. Increased proteolysis due to phosphorylation has been described for other proteins, including p27 and eNOS [35,36]. However, in these cases the differential stability of the phosphorylated form of the protein is a normal feature of the system and not inducible by drug treatment. The mechanism by which HCQ causes increased degradation of phospho-S6 is not known, but a reasonable hypothesis would be that HCQ might cause the induction of a particular E3 ligase that recognizes phospho-S6. Further work in this area may be able to elucidate this mechanism.

## Supporting Information

S1 File p62 expression level in RCC. RCC cells were cultured in the absence (C) or presence of either 10 μM RAD001 (R) or 75 μM HCQ (H) for two days, and Western analyses was used to measure the levels of p62. The average value was calculated from between 4 and 6 independent experiments depending on the cell line. The relative expression value is assigned as Arbitrary Units obtained by calculating the ratio of test sample divided by a control.
(PDF)

S2 File Autophagy markers in HCQ-treated cells. 769-P cells were treated with either RAD001 or HCQ for the indicated amount of time and examined for the LC3, beclin 1, and p62. Increased accumulation of LC3(II) was shown by HCQ treatment
(PDF)

S3 File Bafilomycin A1 and Spautin-1 effects on the S6 phosphorylation. **Figure A.** 769-P cells were treated with either bafilomycin A1 or spautin-1 for 0–48 hours and examined for the indicated proteins. **Figure B.** 769-P cells were seeded on 96-well plates at a concentration of 1x10^4^ cells/well in 16 wells and were treated with nothing (Con), 50 nM bafilomycin A1 (Baf), or 10 μM spautin-1 (Spa), or 75 μM HCQ After 48 hours, cell growth was measured using a MTT assay. Error bars show standard deviation, and the letter at top indicates statistically significant differences between columns with different letters (P<0.05, ANOVA with Tukey post-hoc test).
(PDF)
