# Supplementary figures and images for: Hydroxychloroquine Destabilizes Phospho-S6 in Human Renal Carcinoma Cells
Source: PLoS One. 2015 Jul 2;10(7):e0131464. doi: 10.1371/journal.pone.0131464 (PMC4489871; doi:10.1371/journal.pone.0131464)

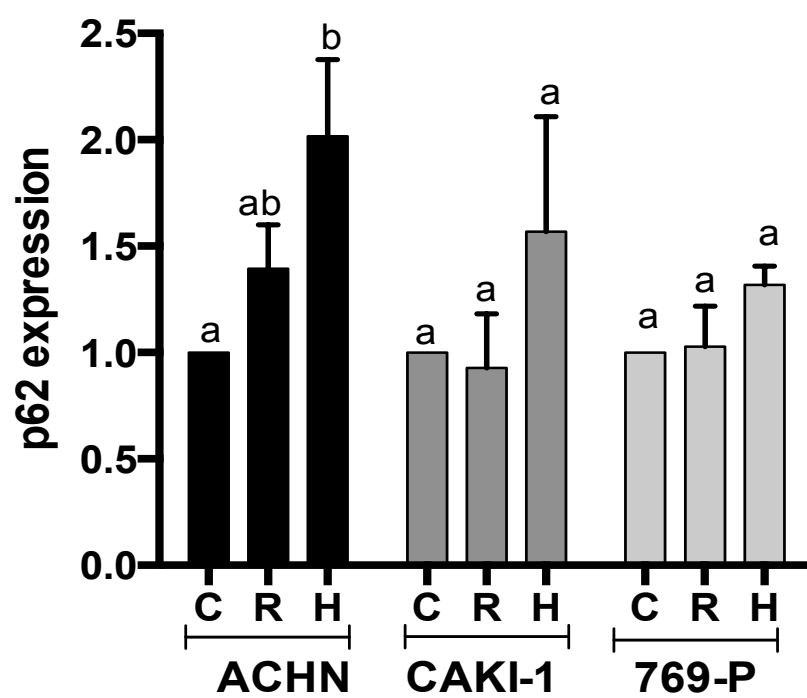

Supplement: S1 File — RCC cells were cultured in the absence (C) or presence of either 10 μM RAD001 (R) or 75 μM HCQ (H) for two days, and Western analyses was used to measure the levels of p62. The average value was calculated from between 4 and 6 independent experiments depending on the cell line. The relative expression value is assigned as Arbitrary Units obtained by calculating the ratio of test sample divided by a control. (PDF) [file pone.0131464.s001.pdf]

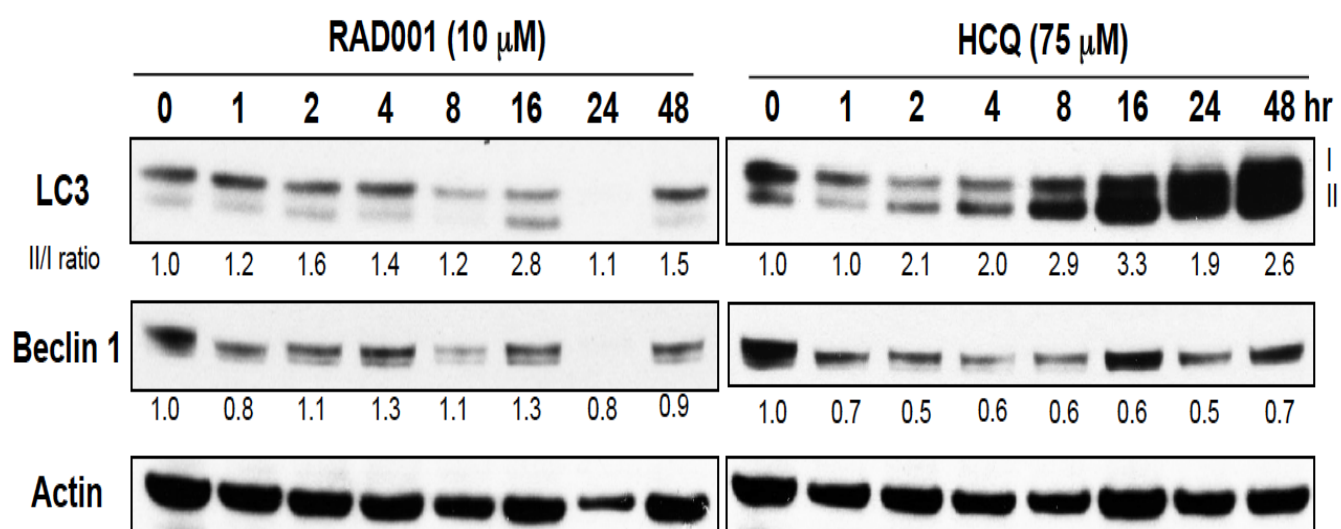

Supplement: S2 File — 769-P cells were treated with either RAD001 or HCQ for the indicated amount of time and examined for the LC3, beclin 1, and p62. Increased accumulation of LC3(II) was shown by HCQ treatment (PDF) [file pone.0131464.s002.pdf]

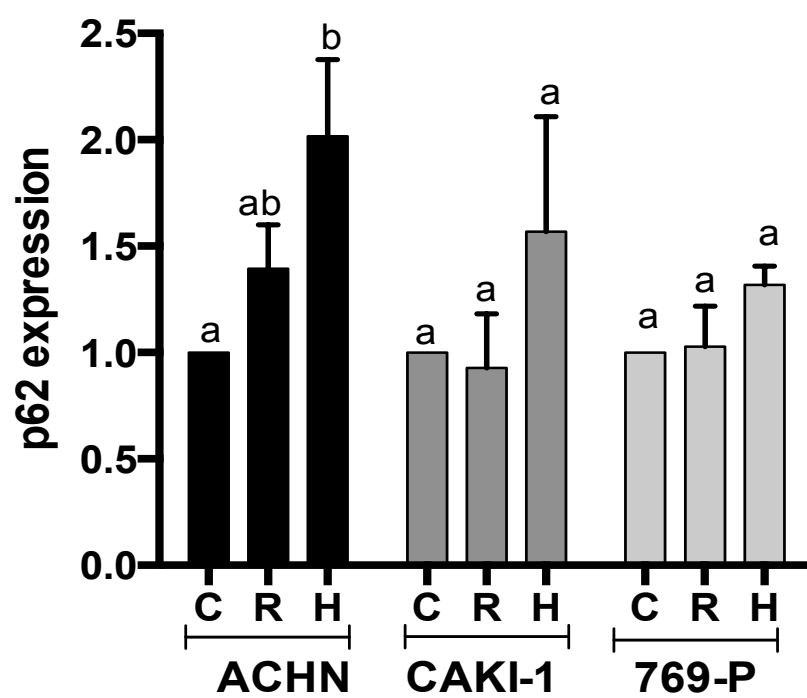

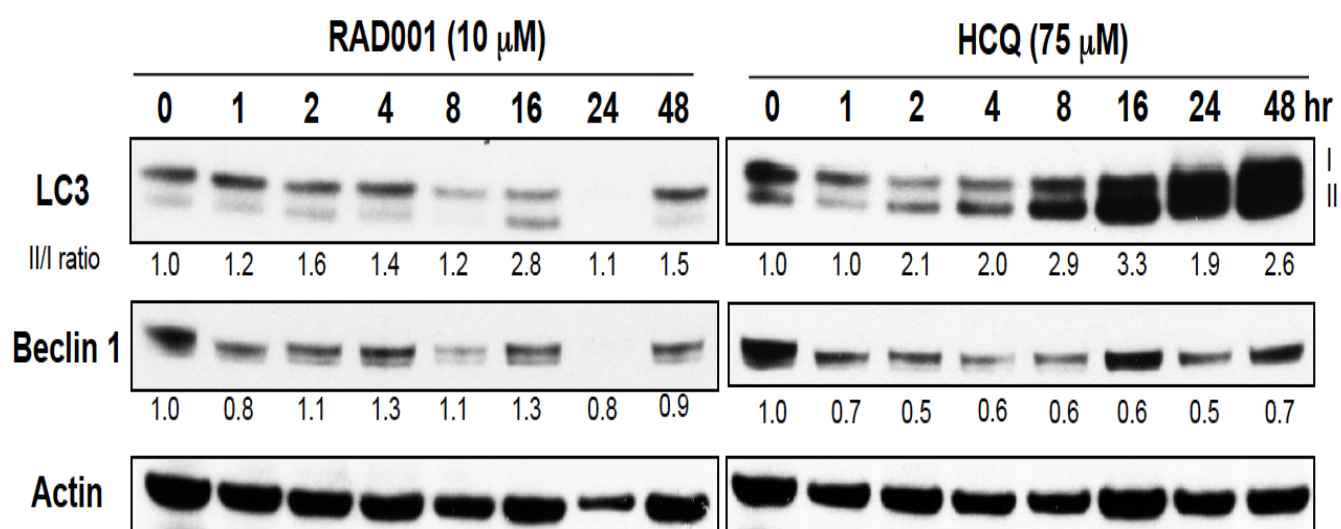

A

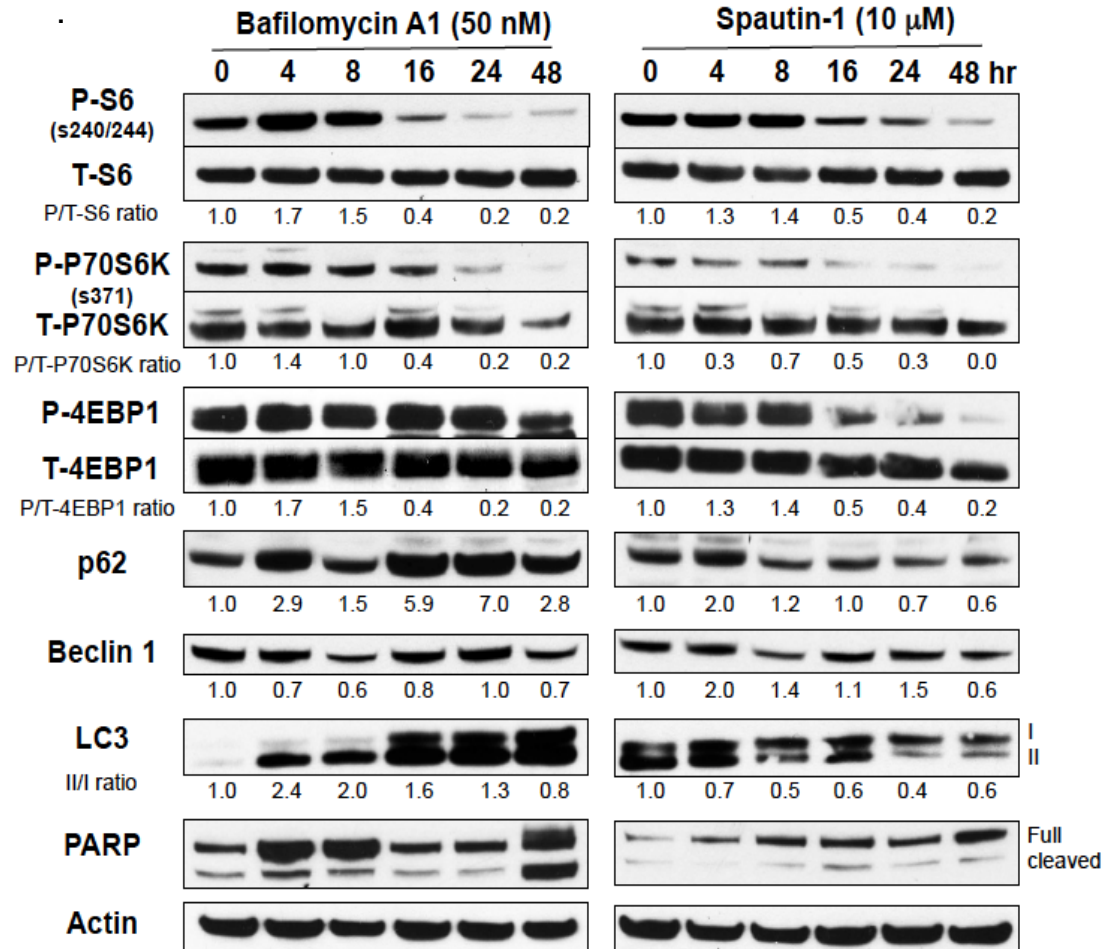

B.

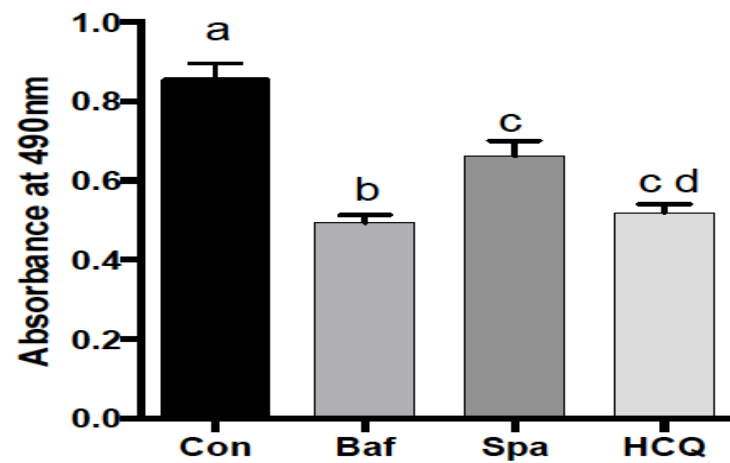

Supplement: S3 File — Figure A. 769-P cells were treated with either bafilomycin A1 or spautin-1 for 0–48 hours and examined for the indicated proteins. Figure B. 769-P cells were seeded on 96-well plates at a concentration of 1x10^4^ cells/well in 16 wells and were treated with nothing (Con), 50 nM bafilomycin A1 (Baf), or 10 μM spautin-1 (Spa), or 75 μM HCQ After 48 hours, cell growth was measured using a MTT assay. Error bars show standard deviation, and the letter at top indicates statistically significant differences between columns with different letters (P<0.05, ANOVA with Tukey post-hoc test). (PDF) [file pone.0131464.s003.pdf]
